# Eco-bio-social research on community-based approaches for Chagas disease vector control in Latin America

**DOI:** 10.1093/trstmh/tru203

**Published:** 2015-01-19

**Authors:** Ricardo E. Gürtler, Zaida E. Yadon

**Affiliations:** a Laboratory of Eco-Epidemiology, Department of Ecology, Genetics and Evolution, Universidad de Buenos Aires-IEGEBA (CONICET-UBA), Buenos Aires, Argentina; b Communicable Diseases Department, Pan American Health Organization, Washington, D.C., USA

**Keywords:** Chagas disease, Community, Eco-bio-social research, Ecohealth, Integrated vector management, Vector-borne diseases

## Abstract

This article provides an overview of three research projects which designed and implemented innovative interventions for Chagas disease vector control in Bolivia, Guatemala and Mexico. The research initiative was based on sound principles of community-based ecosystem management (ecohealth), integrated vector management, and interdisciplinary analysis. The initial situational analysis achieved a better understanding of ecological, biological and social determinants of domestic infestation. The key factors identified included: housing quality; type of peridomestic habitats; presence and abundance of domestic dogs, chickens and synanthropic rodents; proximity to public lights; location in the periphery of the village. In Bolivia, plastering of mud walls with appropriate local materials and regular cleaning of beds and of clothes next to the walls, substantially decreased domestic infestation and abundance of the insect vector *Triatoma infestans*. The Guatemalan project revealed close links between house infestation by rodents and *Triatoma dimidiata*, and vector infection with *Trypanosoma cruzi*. A novel community-operated rodent control program significantly reduced rodent infestation and bug infection. In Mexico, large-scale implementation of window screens translated into promising reductions in domestic infestation. A multi-pronged approach including community mobilisation and empowerment, intersectoral cooperation and adhesion to integrated vector management principles may be the key to sustainable vector and disease control in the affected regions.

## Introduction

With 8–12 million infected people, Chagas disease ranks among the most important infectious diseases in the Americas in terms of disability-adjusted life-years.^1,2^ The disease is caused by the protozoan *Trypanosoma cruzi* which infects at least 150 mammalian species and is mainly transmitted by blood-sucking triatomine bugs.^3^ Chagas disease has strong links to rural poverty, poor quality housing, subsistence economies, and marginalized populations.^2^ Increased international migration from Latin America to high-income countries has expanded the distribution of human Chagas disease to the global scale through transfusional and vertical transmission.

Of the approximately 140 species of triatomine bugs identified, only a handful has adapted to live in houses and blood-feed on humans and domestic animals.^3^ These vector species are the most successful in terms of abundance and parasite transmission, and are the main targets of vector control programs: *Triatoma infestans* in the southern cone countries; *Rhodnius prolixus* in the northern section of South America and formerly in several countries of Central America; *Panstrongylus megistus* in Brazil; *Triatoma dimidiata* in Central America, southern Mexico, Colombia and Ecuador. However, a much longer list of sylvatic triatomine species may also infest peridomestic structures and eventually establish domestic colonies, contact humans and transmit *T. cruzi*. These species are hard to control by classical strategies and include *Triatoma brasiliensis* in northeastern Brazil;^4^ *Rhodnius pallescens* in Central America;^5^ and *Rhodnius ecuadoriensis* in Ecuador and northern Peru,^6^ among others. Yet another group of triatomine species may be in the process of adapting to domestic habitats, pushed by large-scale changes in land use.

In the absence of vaccines, the primary prevention of human *T. cruzi* infection has relied almost exclusively on residual insecticide spraying campaigns conducted by vertically-structured vector control programs starting in the late 1940s. The only two drugs available for treatment since the early 1970s were until recently restricted to the acute or recent stage of infection.^7^ A series of intergovernmental initiatives created in the 1990s coordinated vector control efforts at regional levels and substantially reduced house infestation indices and disease incidence in several countries of South and Central America.^3,8^ Progress was uneven, and what was effective in some areas (e.g. Brazil, Uruguay and Chile) had lower impact elsewhere, such as in the case of *T. infestans* in the Gran Chaco region of Argentina, Bolivia and Paraguay.^9,10^ For *R. prolixus* in South America,^11,12^ *T. dimidiata* in Central America and Mexico,^8^ and *T. brasiliensis*,^4^ the goal of vector elimination is not feasible and control programs have to grapple with the recurrent nature of house invasion and recolonisation, vector surveillance and insecticide treatment.

Centralized vertical control programs are ill-equipped to deal with these recurrent tasks, especially in resource-constrained rural areas with low-density, dispersed human populations that are difficult to access, resulting in high operating costs. Furthermore, the operational capacity of most Chagas disease vector control programs has been seriously compromised by the health care reforms (decentralization) started in the early 1980s,^9^ in a context of unstable developing economies and sociopolitical situations. This problem was further compounded with the low political priority assigned to Chagas disease by many of the affected countries. In the absence of permanent vector suppression, the question of how to sustain the open-ended vector surveillance phase still is unresolved.

Failure to achieve the stated program goals of vector elimination led to 1) the search for more sustainable, integrated control strategies that address ecological and social determinants of parasite transmission; 2) more emphasis on participatory frameworks and intersectoral cooperation (to ensure sustainability); 3) the application of integrated vector management (IVM) principles (virtually absent in Chagas disease vector control).^13^ Rural housing programs and other environmental management measures^14^ were rarely considered part of an integrated Chagas disease control strategy and reliance on insecticides has stayed approximately the same since they first came into use more than 60 years ago.

Building upon a multi-country research initiative for targeted dengue vector interventions in Asia,^15^ in 2007 the Special Programme for Research and Training in Tropical Diseases (TDR/WHO) and the Ecosystem and Human Health Program of Canada's International Development Research Centre (IDRC) launched a research and capacity building partnership named ‘Innovative community-based ecosystem management interventions for improved dengue and Chagas disease prevention in Latin America.’ This initiative aimed to improve dengue and Chagas disease vector control through innovative interventions based on sound principles of community-based ecosystem management (ecohealth),^16^ IVM^13^ and interdisciplinary analysis. The initiative's goals were to achieve a better understanding of ecological, biological and social (eco-bio-social) determinants of vector abundance and parasite transmission,^15,17^ and to develop, implement and evaluate intersectoral, community-centred interventions directed at reducing transmission risks.

Here we provide an overview of the three research projects on Chagas disease vector control interventions whose results are published in this issue.^18–20^ We briefly review the characteristics of study sites and methods, describe the main results obtained before implementing carefully designed control interventions and assess the initial impacts of these interventions in search of common patterns. This cross-site comparison is intended to bring together the main outcomes of the three projects and their repercussions for Chagas disease vector control beyond the specificities of each site and vector species implicated.

## Methods

### Study area and design

The studies were carried out in rural areas of Bolivia, Guatemala and Mexico with clearly established threats of transmission of *T. cruzi* in domestic and peridomestic habitats (Table 1). Although the three projects did not constitute a multi-site study, they shared the same general scope in overall study design and methods.^18–22^

**Table 1.** Summary description of study design and main findings at baseline of the three research projects on community-based approaches for Chagas disease vector control

Reference	Research sites (village/municipality; department; country)	Baseline study design and sample size	Risk factors for house infestation^a^	Additional findings
Dumonteil et al. 2013^21^	Bokoba, Teya and Sudzal; Yucatan; Mexico.	Simple random survey of 20% of all houses; 346 houses in the three villages.	Most important factors: Number of dogs Keeping chickens in a coop Proximity to the periphery of the village Cleaning of peridomestic areas Storage of firewood indoors Moderately important factors: Peridomestic rock piles Proximity to public lights	Baseline house infestation: Sudzal: 14.3% (10/70) Bokoba: 8.5% (9/106) Teya: 20.5% (27/132) Mean: 14.9% (46/308) Very limited house colonization; extensive transient infestation. Storage of firewood and cleaning of peridomicile associated inversely with house infestation.
Bustamante et al. 2014^22^	Comapa and Zapotitlán municipalities; Jutiapa; Guatemala.	Two-stage cluster sampling including 32 villages and 472 houses.	Most important factors: Number of dogs Rodent infestation Condition of interior wall plaster Dirt floor Tiled roofing Coffee tree	Baseline house infestation: Timed searches: 25.4% (120/472) Householders' bug collections: 30.2% (144/477) Houses infested with ≥1 infected bug: 30.8% (40/130) House infestation by rodents: 59.8% (149/249)
Lardeux et al. 2015^18^	Eje Pampa and Lagar Pampa; Cochabamba; Bolivia. Palmarito and La Brecha; Santa Cruz de la Sierra; Bolivia.	Complete enumeration of all listed houses: two villages with 80 and 87 houses (Chaco region); two villages with 123 and 95 houses (Valleys region).	Statistically significant factors in the Chaco region: Cracked walls Chickens nesting indoors Presence of beds in the room Poor roof condition Statistically significant factors in the Valleys region: Bags stored hanging on walls	Baseline domestic infestation: Chaco region: 95% (76/80) and 92% (76/83) Valleys region: 28% (13/47) and 35% (15/43) Houses with ≥1 bug-infested bed: Chaco region: 43.9% (141/321) and 46.8% (110/235) Valleys region: 1% (1/74) and 3% (1/38)

^a^ Domestic infestation assessed by householders' bug collections over a one-year period (Mexico); timed manual searches in domestic and peridomestic habitats and householders' bug collections (Guatemala); manual searches in domestic and peridomestic habitats (Bolivia).

Local stakeholders, including health departments, participated in the projects from their onset. All projects included a baseline situational analysis (phase I) with common objectives: 1) To assess three key ecosystem dimensions affecting the presence and abundance of Chagas disease vectors in human dwellings and their interactions: vector biology/ecology and related environmental factors; the social, cultural, economic and community context; vector control program organisation and functioning; 2) To analyse the relative importance of eco-bio-social factors associated with different levels of vector presence and abundance; 3) Based on the situational analysis, to identify and design specific interventions appropriate to the domestic and peridomestic environments through a community-based, participatory process. Consideration of the specificities of each study site and triatomine species led to a range of interventions and their combinations. Phase II included three objectives: 1) to implement and monitor the designed interventions; 2) to evaluate their effectiveness; 3) to recommend community-centred intersectoral ecosystem intervention approaches.

### Design of interventions and impact assessment

The research teams used the outcomes of the situational analysis to design specific intervention packages tailored to each system through a participatory process involving multiple stakeholders and the affected communities (Table 2). Their cultural practices, concerns or priorities were considered in the design and implementation of the interventions. An explicit goal was to achieve community empowerment. The costs of interventions were partially or fully covered by the projects.^18–20^

**Table 2.** Summary description of the main results of the implementation of vector control interventions in the three research projects

Reference	Research sites (village/ municipality; department; country)	Interventions			Midterm or endpoint results
		Village	Intervention type	Number of houses intervened	
Waleckx et al. 2015^20^	Bokoba, Teya and Sudzal; Yucatan; Mexico.	Teya	Window screens	702 houses	Screen cost per house: US$35. Moderate to strong participation of local communities and stakeholders. Significant midterm reduction of indoor versus outdoor house infestation when window screens were present. High potential for sustainable reductions in human-vector contact rates, including triatomines and mosquitoes.
		Sudzal	Window screens Education about cleaning of chicken coops (poultry husbandry).	416 houses	
		Bokoba	Control	570 houses	
De Urioste-Stone et al. 2015^19^	Comapa municipality; Jutiapa; Guatemala.	Nine intervened and nine control villages.	Full-coverage insecticide spraying including tiled roofs and all walls. Participatory education on Chagas disease, risk factors and training in rodent control. Organic waste management combined with productive activities.	18 villages and 429 houses	Effects on domestic infestation: Pre-intervention: Treatment (n=194): 19.3% ±10.2%; Control (n=199): 19.0%±9.0% Post-intervention: Treatment (n=194): 7.9%±7.0%; Control (n=199): 5.9%±5.8% Endpoint effects: no statistically significant differences between treatment and control. Reduced relative odds of early-stage bug infection in treated versus control villages. Effects on *Rattus rattus* and *Mus musculus* infestation: Post-intervention rat infestation significantly reduced: Treatment: 8.4% (16/190) Control: 15.6% (30/192) Post-intervention mouse infestation not significantly reduced: Treatment: 33.2% (63/190) Control: 39.6% (76/192) Sustainability closely related to stakeholders' cooperation.
Lardeux et al. 2015^18^	Eje Pampa and Lagar Pampa; Cochabamba; Bolivia. Palmarito and La Brecha; Santa Cruz de la Sierra; Bolivia.	Intervention villages: Eje Pampa and Palmarito Control villages: Lagar Pampa and La Brecha	Wall coating with a standardized mud mixture. House cleaning including beds and objects on walls. Removal of animals from houses, especially poultry.	Eje Pampa: 42 houses. Pamarito: 75 houses.	Midterm effects on domestic bug abundance and prevalence of domestic infestation: Chaco region: both outcome measures were significantly lower in the treated village. Valleys region: similar decreasing trends in all indices of house infestation, no significant differences detected. Limited sustainability because Chagas disease is not perceived as a health risk.

In Guatemala, the intervention package was based on the PRECEDE-PROCEED model for situational analysis, implementation and intervention evaluation.^19,22^ The package integrated the following: 1) educational workshops; 2) improved insecticide spraying procedures with full coverage of tiled roofs and walls; 3) participatory training workshops and implementation of rodent control activities; 4) multisectoral training in organic waste management and productive household activities; 5) a participant-based reflective process.^19^

In Bolivia, the intervention package included low-cost housing improvement techniques and community participation in two intervention versus two control villages.^18^ The goals were to eliminate refuges for bugs in the walls through plastering the surface with appropriate local materials; to promote house cleaning activities directed at beds and objects next to the walls; to remove chickens and dogs from human sleeping quarters, over a 6-month period.^18^ To promote community participation the research team used a multi-pronged strategy based on interpersonal communication, community mobilization, lobbying at community and regional levels, and supported advertising.

In Yucatan (Mexico), the research team conducted a series of meetings with multiple stakeholders to review the main findings of phase I,^21^ proposed the installation of window screens as a barrier against triatomine bugs in two intervention communities, and implemented the interventions in more than 800 houses.^20^ Education workshops addressing management and cleaning of chicken coops were also implemented in one of the villages.

The main outcomes measured included the degrees of acceptance, support and community participation throughout the intervention process. Control and intervention groups were compared in terms of domestic infestation indices at baseline, in a midterm evaluation and at the end point. Measurement of knowledge, attitudes and practices regarding Chagas disease, risk factors and related issues were conducted at set time points. The impacts of interventions were assessed initially within six months of finishing their implementation,^18–20^ and end-point assessments were planned for late 2014.

## Results

### Cross-site findings

Pre-intervention domestic infestation levels combined with bug infection prevalence suggested substantial risks of domestic transmission of *T. cruzi* in the three study sites (Table 1). Despite the fact that official insecticide spraying operations had been conducted in the Bolivian and Guatemalan study areas a few years before, baseline house infestation levels were comparable with active vector-borne transmission as indicated by vector, dog or rodent infection rates with *T. cruzi*. Vector control programs in Mexico traditionally have not conducted systematic insecticide sprays in response to the domestic catch of *T. dimidiata* or any other triatomine bug. The status of transmission was rather uncertain in Yucatan, where the house-invading bugs were infected with *T. cruzi*, domestic colonization was transient and the rates of vector-human contact apparently were more sporadic than in the other study sites. Unpublished data collected locally indicated that the seroprevalence of human *T. cruzi* infection in Bolivia was an order of magnitude higher than in Yucatan.

The three projects used multivariate statistical methods to identify the key factors explaining variations in domestic infestation.^18,21,22^ These factors were related to housing quality, characteristics of peridomestic habitats, availability of domestic animal hosts and location effects (Table 1). Housing quality attributes included the occurrence of cracked walls, earthen floors, tiled roofing (only in the Guatemalan study), bags and objects on the walls (for storage purposes). Relevant aspects of peridomestic habitats included the presence of chicken coops or nests, rock piles and coffee trees. Host availability included the presence and abundance of domestic dogs, chickens and rodents. Proximity to public lights and location in the periphery of the village suggested that house invasion by *T. dimidiata* in Yucatan originated from surrounding sylvatic habitats which initially dispersed towards public light sources.

The implementation time for interventions varied from 9 months in Bolivia and Guatemala to 12 months in Mexico. Midterm assessments of intervention effects indicated sizable reductions in domestic infestation and bug abundance in Yucatan and Bolivia, and reductions of rat infestations, human- and rodent-vector contact and domestic bug infection in Guatemala (Table 2).^18–20^

## Discussion

The research projects covered two different model systems of house infestation by triatomine bugs: one in which there is stable domestic and peridomestic colonization (*T. infestans* in Bolivia and *T. dimidiata* in Guatemala),^18,19,22^ and one in which house invasion is seasonal and domestic colonization appears to be transient with occasional human-vector contact (*T. dimidiata* in Yucatan, Mexico).^20,21^ Baseline domestic infestation levels ranged from 14.9% (46/308) in the Mexican Yucatan region to 95% (76/80) in the Bolivian Chaco region, as determined by different methods, and in most cases are expected to underestimate the actual infestation.^23^ House infestations in Bolivia and Guatemala (as determined by timed manual searches) implied that there were established vector colonies in domestic and peridomestic habitats, whereas ‘domestic infestation’ in Yucatan actually represented transient infestations or house invasions detected by householders and notified to the local health post. Householders' bug collections are appropriate for continued domestic vector surveillance and corroborated that in Yucatan, house invasion was seasonal and stable across years.^21^

The three study locations were rural and differed in house density, infrastructure, degree of remoteness, resource constraints and history of insecticide spraying. Housing quality in Yucatan was substantially better (owing to extensive housing development plans during recent decades) than in Bolivia and Guatemala, households were much less isolated and poverty levels were apparently lower. For example, Teya in Yucatan had street signs and public lights. Conversely, rural villages in Bolivia were very poor, especially in the Chaco region. The Bolivian and Guatemalan studies produced strong evidence of vector-borne transmission of *T. cruzi* in domestic habitats, despite the fact that both areas had been sprayed with pyrethroid insecticides several times over the previous years and no local pyrethroid resistance was detected. In contrast, transmission threats were seasonally recurrent in Yucatan. Current routine operations against *T. dimidiata* in Central America rely on indoor (domestic) residual spraying with insecticides whereas full-coverage of domestic and peridomestic sites is recommended for *T. infestans*.

The initial situational analysis provided a deeper understanding of the study systems, guided the design and successful implementation of control interventions in community-centred efforts and gained support from several stakeholders. The key factors explaining variations in domestic bug infestation across study sites may be grouped according to housing quality, availability of animal hosts and location effects. Housing quality affected the availability of refuges for bugs in human sleeping quarters (i.e. cracked walls, earthen floors, tiled roofing, bags and clothes hanging on walls) and in peridomestic habitats (chicken coops or nests, rock piles and coffee trees).^18,21,22^ These results confirm previous findings that housing design and construction materials combined with their degree of maintenance affect the susceptibility of a house to bug invasion and subsequent development of domestic bug colonies.^24–28^ More specifically, the surface structure of indoor walls and the occurrence of thatched roofs were significant predictors of domestic infestation and abundance for the four major domestic vectors of *T. cruzi* elsewhere.^11,25,26,29–32^

However, the specificities of how housing quality affects domestic infestation vary between settings and triatomine bug species. Earthen floors are highly important for *T. dimidiata* which displays singular camouflage behaviour with dirt, unlike other triatomines.^33^ In the past, however, the predicted effects of earthen floors were not always supported by multivariate analyses of relatively large numbers of houses.^31,34^ Using a multi-model inference frame and an extensive database, the Guatemalan study documented that earthen floors and wall plaster condition were of high relative importance for domestic infestation.^22^ The Bolivian project emphasized the role of bags, objects on the walls and beds as important refuges for *T. infestans*.^18^ Beds were also a prime bug habitat in the Argentine Chaco:^25^ bugs from beds have immediate access to a human blood meal and are frequently infected with *T. cruzi*. Regular cleaning of beds and removing objects and clothes from the walls were therefore predicted to substantially decrease domestic infestation and bug abundance in the Bolivian study sites. The midterm assessment of intervention effects demonstrated substantial impacts on both outcome measures overall and in the specific bug habitats, especially in the Chaco region of Bolivia.^18^

Following a pilot trial showing that window screens greatly reduced house invasion by *T. dimidiata* in Yucatan,^35^ the large-scale situational analysis corroborated these results and provided another entry point for intervention.^21^ The intervention package addressed the concerns of the affected communities and stakeholders and gained their full support. It was successfully implemented at a village-wide scale and showed promising reductions in domestic infestation by *T. dimidiata*.^20^ The planned end-point assessment will take into account seasonal effects on house invasion and provide concluding evidence on intervention impacts.

The three studies highlighted the relevance of peridomestic structures as breeding or resting sites and sources of the triatomines that invade human sleeping quarters despite the fact that the study settings were notably different. Chicken coops or nests^27^ and poorly-built goat or pig corrals^36^ with many refuges and hosts were productive sources of *T. infestans* and other triatomines in the Argentine Chaco before and after residual insecticide spraying. These habitats provide better conditions and resources for the triatomine bugs than sylvatic habitats where the local hosts may not be available throughout the year. Improvement of goat corrals^37^ and other peridomestic structures^38^ may therefore reduce local infestations and contribute both to integrated vector control and enhanced livestock or poultry production, because high-density triatomine colonies also cause significant blood loss and anaemia. Implementing these environmental management measures indispensably requires the participation of householders and rural development agencies.

The Yucatan project suggests that regular clean-up of peridomestic areas may have mixed effects on domestic infestation by *T. dimidiata* depending on other unidentified factors.^21,35^ Cleaning peridomiciles may both reduce peridomestic bug habitats and (peri)domestic infestation, as observed in Costa Rica,^38^ but it may also facilitate the invasion of domestic premises by sylvatic triatomines that are no longer deflected by suitable peridomestic sites. In line with these findings, elsewhere in Guatemala the plastering of walls combined with insecticide spraying modified substantially the relative abundance of domestic and peridomestic *T. dimidiata* compared with insecticide spraying alone: reduction in the abundance of domestic refuges was followed by a decrease in the abundance of domestic bugs and an increase of peridomestic bugs.^28^ These results highlight the need for integrated environmental management of domestic and peridomestic habitats.

Host availability includes the presence or abundance of domestic and synanthropic hosts as blood meal sources. The key roles of chickens and dogs in the domestic ecology of Chagas disease vectors have been corroborated across multiple settings and triatomine species.^18,21,22,25,27^ Blood meal identification tests also demonstrated that chickens, dogs and humans are major hosts of domestic and peridomestic *T. dimidiata* and *T. infestans* in the study areas and elsewhere.^22,39,40^ The frequent practice of keeping chickens indoors for protection in rural areas with a subsistence economy notoriously resists change,^18^ and so does the habit of letting domestic dogs wander freely and share domestic premises with house-dwellers in poverty-stricken regions. These challenges still need to be addressed effectively.

The additional contribution of house mice and rats to domestic bug infection with *T. cruzi*, combined with the strong association between *T. dimidiata* and rodent infestations, are novel findings with clear implications for the design of innovative, integrated vector control strategies. Although synanthropic rodents were infected by *T. cruzi* in various settings,^41,42^ their role as (peri)domestic reservoir hosts and the effects of rodent control measures on bug infestation and parasite transmission had not been assessed. The Guatemalan project corroborated large house infestation rates with rodents,^31^ and revealed strong links between rats and mice in walls and tiled roofs, infestation with *T. dimidiata* and *T. cruzi* infection.^22^ These results paved the way for a novel community-based rodent control program that received immediate acceptance and was fully implemented by the affected communities. The endpoint assessment showed significant reductions in rodent infestation or abundance and in the prevalence of *T. cruzi* infection among early-stage nymphs.^19^ It remains to be seen whether reducing the abundance of rodents will decrease house infestation with *T. dimidiata* and bug infection over time, and how this may impact on human-vector contact rates. If rodent numbers have an additive effect on bug population growth and infection, as domestic dogs do in northern Argentina,^43^ the risk of bug infection is predicted to decline with effective rodent control, as the impact assessment suggests. However, the direct effects of reducing the abundance of rodents on domestic bug infestation may not be obvious in the presence of several other domestic hosts (humans, dogs and chickens) to which the bugs may shift. Furthermore, sylvatic *T. dimidiata* may still invade the premises and confound the relationship between domestic bug infestation or infection and rodents.

Spatial risk factors for domestic infestation by *T. dimidiata* were identified in Yucatan, where proximity to the periphery of the village apparently favoured house invasion from the surrounding sylvatic habitats.^21^ These results are consistent with other risk factor analyses of (peri)domestic infestation by *Triatoma pallidipennis* and *R. prolixus*, which showed that the spatial characteristics of residences or their distance to specific vegetation harbouring bug colonies need to be considered more closely.^12,44^

Other risk factors usually associated with domestic triatomine infestations were found to be relatively not as important as in many other studies, including the number of people resident in the household^27,32,45^ and household insecticide use.^25,27^ Because of the close links between domestic triatomines and humans as main blood meal sources,^40,46^ the absence of statistically significant effects of household size on domestic infestation needs to be interpreted with caution.

Future efforts to identify relevant risk factors for house infestation may take advantage of current results and explore further avenues. Measuring the impact of control interventions on additional response variables (i.e. host-vector contact and vector blood-feeding rates;^46,47^ combined measures of bug abundance and infection at well-defined habitats; domestic host infection^10^) is needed to translate reductions in house infestation into transmission risks and human infection. Improved estimates of local bug abundance (rather than presence/absence estimates) are needed for assessing habitat suitability and transmission risks. Knowledge on the key sources of triatomine bugs in domestic, peridomestic and sylvatic habitats and their relative habitat suitability may be used for targeted vector control and risk stratification,^48^ as with *R. prolixus* and *Attalea butyracea* palm trees in Venezuela.^12^ The combined use of site-specific timed manual searches and bug sensing devices for continuous monitoring of infestations may give valuable information on the different demographic processes involved in house (re)infestation. The frequent finding of peridomestic colonies of *T. dimidiata*^49^ suggests that IVM (including modification of key peridomestic habitats combined with carefully administered peridomestic insecticide sprays) may be justified in areas with recurrent domestic infestation and transmission risks. More investigations including the social determinants of health, house infestation and domestic transmission of *T. cruzi* are needed.^50^

Community-based vector control interventions with an emphasis on sustainability demand additional follow-up time and efforts for a full appraisal. Some crucial questions include: How can sustainability be evaluated and how long is needed to do so? How feasible is it that the communities will continue with the interventions on their own and that such interventions will be incorporated into local control programmes? Window screens also addressed a major local concern (mosquito nuisance) and may also help prevent dengue in Yucatan,^51^ whereas rodent reduction was welcome in Guatemala because of its effects on food production and storage. These collateral benefits^52^ may be crucial for the sustainability of community-based control interventions against Chagas disease vectors.

### Conclusions

The three projects identified quasi-stable determinants of domestic infestation that most likely underlie the recurrent nature of house recolonisation by triatomine bugs. Improving the quality of rural housing and living conditions results in healthier homes with direct repercussions on community health beyond Chagas disease. Partnering with stakeholders working on other diseases or disciplines may help address problems that have similar causes. A multi-pronged approach including community mobilisation and empowerment, intersectoral cooperation and adhesion to IVM principles may be the key to sustainable vector and disease control in the affected regions.
